# Convergent-beam attosecond x-ray crystallography

**DOI:** 10.1063/4.0000275

**Published:** 2025-01-09

**Authors:** Henry N. Chapman, Chufeng Li, Saša Bajt, Mansi Butola, J. Lukas Dresselhaus, Dmitry Egorov, Holger Fleckenstein, Nikolay Ivanov, Antonia Kiene, Bjarne Klopprogge, Viviane Kremling, Philipp Middendorf, Dominik Oberthuer, Mauro Prasciolu, T. Emilie S. Scheer, Janina Sprenger, Jia Chyi Wong, Oleksandr Yefanov, Margarita Zakharova, Wenhui Zhang

**Affiliations:** 1 Center for Free-Electron Laser Science CFEL, Deutsches Elektronen-Synchrotron DESY, Notkestr. 85, 22607 Hamburg, Germany; 2 The Hamburg Center for Ultrafast Imaging, Luruper Chaussee 149, 22761 Hamburg, Germany; 3 Department of Physics, University of Hamburg, Luruper Chaussee 149, 22761 Hamburg, Germany

## Abstract

Sub-ångström spatial resolution of electron density coupled with sub-femtosecond to few-femtosecond temporal resolution is required to directly observe the dynamics of the electronic structure of a molecule after photoinitiation or some other ultrafast perturbation, such as by soft X-rays. Meeting this challenge, pushing the field of quantum crystallography to attosecond timescales, would bring insights into how the electronic and nuclear degrees of freedom couple, enable the study of quantum coherences involved in molecular dynamics, and ultimately enable these dynamics to be controlled. Here, we propose to reach this realm by employing convergent-beam x-ray crystallography with high-power attosecond pulses from a hard-x-ray free-electron laser. We show that with dispersive optics, such as multilayer Laue lenses of high numerical aperture, it becomes possible to encode time into the resulting diffraction pattern with deep sub-femtosecond precision. Each snapshot diffraction pattern consists of Bragg streaks that can be mapped back to arrival times and positions of X-rays on the face of a crystal. This can span tens of femtoseconds and can be finely sampled as we demonstrate experimentally. The approach brings several other advantages, such as an increase in the number of observable reflections in a snapshot diffraction pattern, all fully integrated, to improve the speed and accuracy of serial crystallography—especially for crystals of small molecules.

## I. INTRODUCTION

Time-resolved serial femtosecond crystallography has opened up the domain of ultrafast structural biology and chemical dynamics.^1,2^ By utilizing intense femtosecond-duration pulses from x-ray free-electron lasers, complete sets of structure factors of crystals of photoactive macromolecules have been recorded at a series of delay times after reaction initiation using an ultrafast optical laser pulse. In this way, for example, the nuclear motions in processes such as photosynthesis in a photosynthetic reaction center,^3^ light sensing in photoactive yellow protein^4^ and rhodopsins,^5,6^ the response of a photoswitchable fluorescent protein,^7^ and DNA repair by photolyase^8,9^ have been observed on femtosecond timescales. The method has also recently been applied to measure the response of a light-sensitive metal-organic framework structure.^10^ Although the number of known photoactive proteins and other molecules is comparatively small, these studies may offer direct insights into chemistry and allow the validation of quantum chemistry codes. The nuclear motions, however, act in response to excited-state electron dynamics initiated by the absorption of a photon. Having much lower mass, the dynamics of electrons occur on even faster timescales. So far, spectroscopic methods have been the only route to investigate these rapid changes.^11^ A real-space visualization of structural dynamics on sub-femtosecond to few-femtosecond time scales is required to improve our understanding of the evolution of the excited system and the coupling of electronic and nuclear motions in molecules. For example, it could enable the observation of the non-adiabatic evolution of dynamics of an excited molecule, where the superpositions of wave packets on the potential-energy surfaces may lead to interferences that may route the electrons and nuclei back to the ground state or to various product states. Tracking the transient electron density could complement measurements of complex ionization dynamics in molecules.^12^ With the recent development of sub-femtosecond duration x-ray pulses with terawatt powers,^13^ it becomes feasible to conduct crystallography in the attosecond regime. Such an undertaking comes with several challenges. High-precision crystallographic measurements are required at resolutions better than the atomic scale of 0.7 Å or so to be able to characterize weak changes of electron density, as is achieved in the field of quantum crystallography^14^ and recently demonstrated in static serial crystallography experiments on rhodamine^15^ and in femtosecond x-ray powder diffraction of charge relocations in terahertz excitation of aspirin crystals.^16^ Reaction initiation with sub-femtosecond precision necessarily demands wavelengths in the deep UV or shorter, such as utilized in attosecond transient absorption spectroscopy.^17^ Path differences between pump and probe beams must be controlled to below about 30 nm. Addressing these challenges would no doubt help further develop and improve the study of time-resolved structures also at longer timescales.

Here, we propose and explore a route based on convergent-beam diffraction, utilizing a highly focused beam, to address some of these challenges. The approach could greatly increase the information content of single snapshot patterns by providing many more Bragg reflections than with a collimated monochromatic beam, and ensuring that most of those are fully integrated, thereby enabling the acquisition of datasets with higher precision or with fewer diffraction patterns. Since the method combines aspects of projection imaging, effects of crystal shape and structure can be inferred and accounted for, improving the accuracy of the dataset. A particularly useful characteristic of convergent-beam diffraction that we examine here is that time can be directly encoded in the angular distribution of diffracted intensities, analogous to the way that chirped-pulse Laue diffraction maps time to the wavelength of reflections as proposed by Keith Moffat.^18^ Furthermore, with high-quality focusing optics, the necessary wavefront control for sub-femtosecond timing should be achievable.

Following a brief overview of serial crystallography and its extension to pink-beam measurements in Sec. II, convergent-beam diffraction of three-dimensional crystals is introduced in Sec. III and compared with Laue diffraction. Two particular schemes are considered: one in Sec. III A where the crystal is located in the focal plane of the x-ray lens, and the other in Sec. III B where it is placed out of focus. In both cases, time can be encoded directly in the diffraction patterns when using a dispersive focusing lens, as explained in Sec. IV. The latter scheme enables highly magnified topograms of the crystal to be obtained, from which the x-ray arrival time can be determined. For visible or UV pump pulses, we must consider that the refractive index of the crystal material is considerably different than for X-rays, leading to different speeds of propagation through the crystal. Furthermore, a higher timing jitter between a UV-visible pump and the x-ray probe is expected than achievable using two-color x-ray generation.^19^ Particular velocity-matching schemes are required, as described in Sec. V. These constrain the orientations of the crystal and pump pulse relative to the x-ray beam that can be used to perform time-resolved crossed-beam topography,^20^ but nevertheless allow diffraction data to be obtained over a span of tens of femtoseconds in a single snapshot pattern while maintaining sub-femtosecond time resolution. We present an experimental test of convergent-beam diffraction on a vitamin B_12_ crystal in Sec. VII using an x-ray lens of very high numerical aperture. In Sec. VIII, we show that our particular experimental geometry would encode time in the diffraction pattern to a resolution approaching 10 as over a range of 12 fs and describe how this can be adjusted.

## II. SERIAL FEMTOSECOND CRYSTALLOGRAPHY

Serial femtosecond crystallography is a method where many snapshot (or still) diffraction patterns are collected, each from an individual crystal that is usually in some random and unknown orientation.^21^ The patterns are indexed and intensities in Bragg peaks are extracted and merged, ideally after first accounting for their partialities.^22^ The method can be compared with powder diffraction, where intensities from many individual crystallites are integrated in the laboratory frame over Debye-Scherrer rings. By recording diffraction instead from each single crystallite, the amalgamation of diffraction intensities from many crystals can be performed in a three-dimensional reciprocal space, in the frame of reference of the crystal lattice rather than the laboratory frame, to obtain the complete set of structure factors of the average three-dimensional crystal. By using femtosecond-duration high-intensity x-ray free-electron laser pulses, exposures may greatly exceed the usual limits set by radiation damage of macromolecular crystals, even at ambient temperatures, by outrunning damage processes.^23,24^ The pulse subsequently destroys the exposed crystal so only a single exposure is possible from each. Serial crystallography is also carried out at synchrotron radiation facilities to acquire data at exposures far below radiation damage limits, with the only limitation being that there is enough signal measured in each diffraction pattern to permit accurate indexing and prediction of the locations of Bragg peaks.^25^ Obtaining a 3D map of structure factors at a desired minimum signal to noise ratio is then a matter of measuring a sufficient number of diffraction patterns, provided that time and sample are available. Serial crystallography is suitable for time-resolved crystallography of dynamical systems triggered by light or by mixing of a reagent, particularly since exposure times are short and there is no need to restore the crystal to the ground state. Following recent developments to analyze diffraction patterns that contain only several Bragg peaks,^26,27^ it has recently been applied to the measurement of small-molecule crystals.^10,15,28,29^

Snapshot diffraction patterns of well-ordered crystals made using a collimated quasi-monochromatic x-ray beam consist mainly of partial reflections. This requires tens of thousands of patterns to be measured to obtain accurate estimates of structure factors.^30^ One way to speed up serial crystallography is to record predominantly fully integrated reflections by utilizing the full “pink beam” spectrum of an x-ray undulator.^31^ This is the serial-crystallographic extension of Laue diffraction. A Laue diffraction pattern (that is, the still diffraction pattern of a three-dimensional crystal obtained with a broad bandwidth) consists of many more reflections than does a monochromatic pattern since each peak in the diffraction pattern is formed by a particular wavelength that obeys the Bragg condition for a given spatial frequency or reciprocal lattice vector of the crystal. Laue diffraction was successfully used for early time-resolved macromolecular crystallography experiments,^32,33^ where the increase in total fluence compared with a monochromatic beam enabled exposure times at a synchrotron radiation facility as short as about 100 ps (Ref. 34), and the increase in the coverage of reciprocal space for a single orientation of the crystal allowed full datasets to be collected from just a few (comparatively large) crystals and crystal orientations. Assuming known lattice parameters, indexing the diffraction pattern entails determining both the Bragg index corresponding to each reflection and the particular wavelength. This is a considerably more challenging problem than indexing a monochromatic pattern, especially for snapshot patterns in serial crystallography, since the assignment of each spot to a reciprocal lattice vector depends both on the scattering angle and the unknown wavelength. The pinkIndexer program indexes Laue patterns by finding a consensus solution from many peaks.^35^ In trials of pink-beam serial crystallography, the full 2% bandwidth of an undulator source at a synchrotron radiation facility resulted in structure determination from only 50 still patterns.^31^ A fourfold reduction of the required number of patterns was achieved with XFEL pulses of 2% bandwidth as compared with monochromatic data collection.^36^ The lower reduction in the number of patterns compared with using synchrotron radiation may be due to the fluctuating spectrum of XFEL pulses, reducing the precision of structure factor estimates. If the spectrum is measured on each pulse and the wavelength corresponding to each peak can be identified, then it may be possible to correct for the spectral weighting of each diffraction peak, but this has not yet been demonstrated. It should also be noted that as the bandwidth increases, the signal to background ratio of peaks is reduced since only a small spectral component contributes to each peak, whereas the background is proportional to the total flux.

A particularly elegant approach for time-resolved data collection, put forth by Moffat,^18^ is to carry out Laue diffraction with chirped x-ray pulses. In a chirped pulse, the instantaneous wavelength varies with time. Identifying the wavelength associated with each Bragg peak, by indexing the pattern, allows diffraction intensities to be assigned to a particular time of occurrence, *T_X_*. Many measurements, such as those in serial crystallography, could then be aggregated to obtain a complete set of structure factors as a function of delay $T=T_{X}-T_{p}$ relative to the arrival of an optical pump pulse at *T_p_*, for example. The time resolution in any such pump-probe experiment is given by the quadrature sum of the duration of the pump pulse, the duration of the probe pulse, and the timing jitter between them. A chirped pulse can potentially reduce the contribution of jitter to this timing error by encoding time in the observable diffraction pattern and allowing the durations spanned by different measurements to overlap so they can be registered to each other. Since the chirped pulses are longer than the short-pulse unchirped pulses needed to achieve a certain time resolution, the chirped pulses may be of lower intensity and thus less destructive.^18^

## III. CONVERGENT-BEAM DIFFRACTION

Given that the Bragg equation λ=2d sin θ has only three variables—the wavelength *λ*, the period of the crystal planes *d*, and the Bragg angle of the reflection *θ*—an alternative to Laue diffraction is to supply a range of angles of incidence that illuminate the crystal. This is the case in convergent-beam crystallography,^37,38^ where the beam is brought to a tight focus by a lens of high numerical aperture (NA) such as a multilayer Laue lens (MLL)^39^ and used to illuminate the sample. Such lenses are sliced from a structure made by layer deposition and can now focus to spots that are as small as the unit-cell dimensions of protein crystals.^40^ Equivalently, the angular extent of the focused beam can exceed the angular separation of Bragg peaks. The convergent illumination vastly increases the number of recorded reflections in a single monochromatic diffraction pattern and ensures that they are fully integrated. Just as Laue diffraction offers a way to encode time using a chirped pulse,^18,41^ we show that time can also be encoded in convergent-beam diffraction patterns with a remarkably high resolution, using two distinct schemes. The approaches are compatible with the spectral properties of x-ray FEL pulses, including newly developed attosecond pulses.

**FIG. 1.**
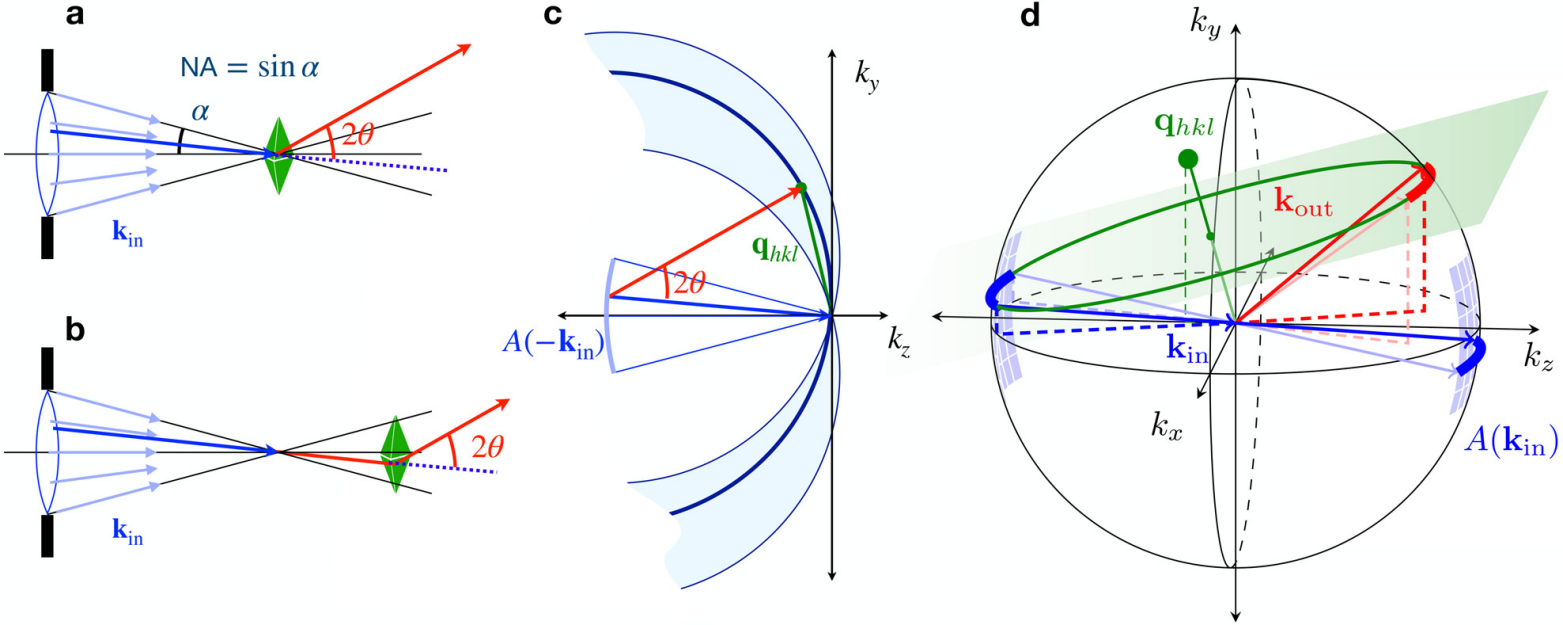
Convergent beam diffraction from a thick crystal in focus (a) occurs within the volume of the crystal intersected by the focused beam. When out of focus (b), diffraction occurs at the positions intersected by the particular $k_{\text{in}}$ vectors (blue) that fulfill the diffracting condition. (c) In both cases, the diffracting condition is determined by the orientation of the Ewald sphere intersecting the reciprocal lattice vector $q_{\text{hkl}}$. (d) The condition is invariant to rotation around the $q_{\text{hkl}}$ vector and so the diffracting and incident vectors describe a circle in the plane normal to and bisecting $q_{\text{hkl}}$. Given the available $k_{\text{in}}$ supplied by the lens aperture $A(-k_{\text{in}})$, these vectors are confined to the surface of a cone, with its apex at the origin, and sweep out arcs (bold lines).

We consider an x-ray beam focused by a lens. The beam near the focus can be described by its angular spectrum of plane waves, whose amplitude and phase are given by the lens pupil function $A(-k_{\text{in}})$, a complex-valued function of wave-vectors $k_{\text{in}}$ that all have magnitude 1/λ. (We use the crystallographic convention that |k|=1/λ and that the magnitude of a reciprocal space vector |q|=1/d, for a spatial period *d*.) The real-space distribution of the field in the focus, a(x), is the synthesis of all the plane wave components, given by the Fourier transform of *A*. Furthermore, the distribution at small distances *z* from the focus can be related to the Fresnel transform of a(x). In the far-field of the focus, which can be reached in just a few hundred Rayleigh lengths, the distribution of the wave-field is again $A(k_{\text{in}})$. This is certainly the case at the plane of the detector where the intensity of the beam diverging from the focus is proportional to $|A(k_{\text{in}}){|}^{2}$. The sine of the semi-angle *α* of the angular distribution of the wave-vectors supplied by the lens is referred to the numerical aperture of the lens, or NA, shown in Fig. 1(a).

The formalism of convergent-beam diffraction is often presented within the context of thin objects that are described by a two-dimensional transmission function, such as in the analysis of ptychography where the object is placed in the focal plane and diffraction is recorded as the objects is scanned in this transverse plane.^42^ The diffraction pattern for each position of the object is given by the Fourier transform of the product of the transmission function with the probe function a(x). For a two-dimensional crystal, for example, the diffraction, therefore, consists of the entire pupil function, *A*, convolved with each diffraction order. If the angular separation of the diffraction orders is small enough, then the pupil functions will overlap and, in the regions of overlap, will thereby interfere, giving the opportunity to determine the relative phases of those orders.^43^ This occurs if the diameter of the pupil, 2NA, exceeds the angular separation λ/d of the orders, which is equivalent to the condition that the width of the focus formed by the unaberrated lens, λ/(2NA), is smaller than the period *d*, stating that that period can be resolved by the lens.

Here, however, we consider a *three-dimensional* object placed at or near the focus and determine the diffraction conditions utilizing the construction of the Ewald sphere and the 3D Fourier spectrum of the object. The Ewald sphere is the two-dimensional manifold in 3D reciprocal space that intersects the spatial frequencies **q** (of the illuminated object) that diffract an incident monochromatic plane wave with wave-vector $k_{\text{in}}$. The Ewald sphere intercepts the origin, q=0, is centered at $-k_{\text{in}}$, and has a radius 1/λ. Since the focused beam incident on the diffracting object can be described as a coherent sum of plane wave components supplied by the lens, the diffraction pattern can be computed as the coherent sum of the diffraction from each component as determined by the intersection of the corresponding Ewald sphere with the object's Fourier spectrum, and after applying the weights $A(k_{\text{in}})$. The complement of Ewald spheres from all wave-vectors provided by the lens fills a volume of reciprocal space, illustrated in Fig. 1(c), that contributes to the convergent-beam diffraction pattern.

For a crystalline object, each reciprocal lattice vector $q_{\text{hkl}}$ within this volume selects an incident $k_{\text{in}}$ according to the Ewald sphere it intersects. The wave-vector of the diffracted beam is given by $k_{\text{out}}=q_{\text{hkl}}+k_{\text{in}}$. Since the range of $k_{\text{in}}$ is two dimensional, there is not just one unique incident wave-vector that satisfies the Bragg condition for a particular reciprocal lattice point as would be the case for Laue diffraction. Indeed, all wave-vectors supplied by the lens that can be formed by rotating $k_{\text{in}}$ around the $q_{\text{hkl}}$ vector will also obey that condition, as seen in Fig. 1(d). Since the magnitudes of $k_{\text{in}}$ and $k_{\text{out}}$ are equal, their average is perpendicular to and bisects $q_{\text{hkl}}$. The rotation of $k_{\text{in}}$ around $q_{\text{hkl}}$ sweeps out a circle in the plane which is normal to $q_{\text{hkl}}$ and intersects the point $q_{\text{hkl}}/2$. The circle [shown in green in Fig. 1(d)] can be found from the solution of the following two equations:

$$k^{2}=\frac{1}{\lambda^{2}};\;\;\;\;\left(k-\frac{q_{\text{hkl}}}{2}\right)\cdot q_{\text{hkl}}=0.$$ (1)

**FIG. 2.**
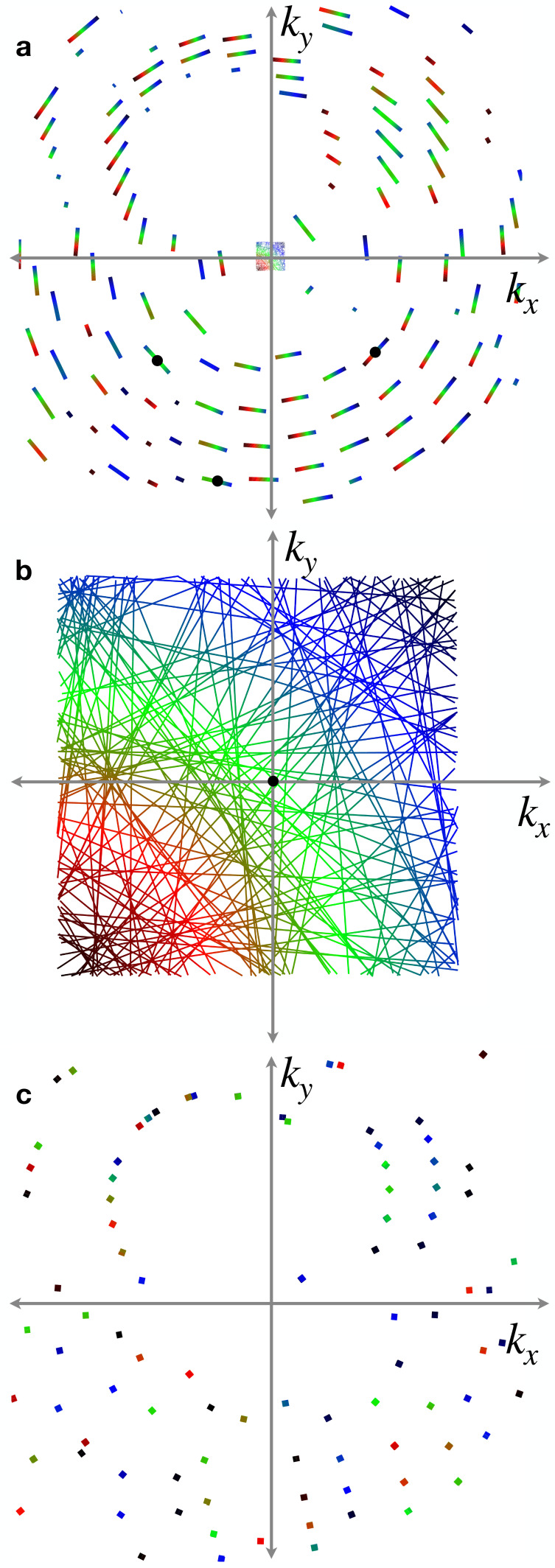
(a) Simulated convergent-beam diffraction pattern showing positions of Bragg streaks to a resolution of 1.5 Å (in the corners of the pattern) at a wavelength of 1 Å with an off-axis lens with a square aperture, NA = 0.035, and for an orthorhombic lattice with unit cell parameters *a* = 9.0 Å, *b* =15.7 Å, *c* = 18.8 Å. The center of the pattern shows the transmitted deficit lines that map out the lens pupil and are shown magnified in (b). The deficit lines and Bragg streaks are colored according to the transverse distance in the lens pupil from the bottom left corner. Black circles indicate the positions of Bragg peaks that would be seen if the NA was reduced to 0.001. (c) Laue diffraction pattern obtained from the same crystal lattice with a relative bandwidth of 25%. In this case the color represents wavelength, ranging from 0.87 Å (dark blue) to 1.12 Å (dark red).

This circle has radius equal to the magnitude $\bar{k}=(1/\lambda )\;\;\text{cos}\;\theta$ of the average vector $\bar{k}=(k_{\text{in}}+k_{\text{out}})/2$ and can be parameterized as

$$k_{p}(\chi )=\frac{q_{\text{hkl}}}{2}+\text{cos}\;\chi \;{\bar{k}}_{0}+\text{sin}\;\chi \;{\bar{k}}_{⊥},$$ (2)

where ${\bar{k}}_{0}$ is initially solved in the plane containing $q_{\text{hkl}}$ and the optical axis (the *z* axis) and ${\bar{k}}_{⊥}$ is set orthogonal to both ${\bar{k}}_{0}$ and $q_{\text{hkl}}$ from their cross product. The range of *χ* for the diffracted $k_{\text{out}}(\chi )=k_{p}(\chi )$ and the incident $k_{\text{in}}(\chi )=k_{p}(\pi -\chi )$ is limited by the extent of the lens pupil. The diffraction due to the reciprocal lattice vector, therefore, consists not of a Bragg peak but a line, which we refer to as a Bragg streak. On a flat detector this is a curved line given by the intersection of the cone of $k_{\text{out}}(\chi )$ with the plane of the detector, but for a small NA the streaks are well approximated by straight lines. The simulated pattern in Fig. 2(a) shows the positions of Bragg streaks for an orthorhombic reciprocal lattice with unit cell dimensions of *a* = 9.0 Å, *b* = 15.7 Å, and *c* = 18.8 Å, chosen to be comparable to those of a pharmaceutical compound. The crystal was taken to be in a non-special orientation, and the pattern was simulated for a lens with NA=0.035, and a wavelength of 1 Å. For this particular orientation, there are 139 Bragg streaks out to a resolution of 1.5 Å.

Each $k_{\text{out}}(\chi )$ Bragg streak in Fig. 2(a) has a corresponding $k_{\text{in}}(\chi )$ “deficit line” that cuts through the pupil as shown in the center of the pattern and magnified in Fig. 2(b). Each deficit line is the line of incident wave-vectors that are diffracted into the corresponding Bragg streak and thus has the same orientation and length as the Bragg streak. The naming is due to the fact that in thick crystals, described by dynamical diffraction theory, the transfer of intensity into the Bragg streak is seen as a loss of intensity in the forward beam, giving a dark line across the direct beam (the pupil function) projected on the detector.^44^ Although for small protein crystals, this loss of intensity is likely not observable, the position of the $k_{\text{in}}(\chi )$ deficit line is nevertheless important because it may map to particular positions across the face of the crystal or delay times, as described below in Secs. III B and IV. The deficit lines can be determined by indexing the pattern, and indeed, the constraint that the $k_{\text{in}}(\chi )$ lines are bounded by the lens pupil can be used to refine the indexing solution (manuscript in preparation). In Fig. 2(b), the $k_{\text{in}}(\chi )$ lines are colored according to the distance from the bottom left corner, and the corresponding $k_{\text{out}}(\chi )$ Bragg streaks are given the same color modulation in Fig. 2(a). Thus, streaks that are oriented diagonally from bottom left to top right change color from red to green to blue whereas streaks in directions orthogonal to that tend to have a single color.

Looking at Fig. 2(b), and considering that the Bragg lines lie on full circles $k_{p}(\chi )$ as depicted in Fig. 1(d), one can appreciate that the number of participating reflections increases with the numerical aperture of the lens. Clearly, if we stop down the aperture, some of the streaks will no longer be included. Reducing the NA to zero, which is the case of a collimated incident beam, would likely only intersect a small number of reflections for this particular lattice, if any, depending on how many deficit lines are intersected, and even then these may only be partial reflections. The gaps between deficit lines indicate that, at least for a perfect crystal, there are many orientations of the crystal for which no lines would be intersected. For a quasi-collimated incident beam with a divergence of 1 mrad only three reflections will be observed for this particular case as shown in Figs. 2(a) and 2(b) by the black circles. (An equivalent result would occur for a crystal with 1 mrad mosaicity illuminated by a collimated incident beam.)

Figure 2(b) also highlights the fact that in convergent-beam diffraction, the Bragg streaks predominantly provide fully integrated intensities across the short width of the Bragg streak (i.e., in the direction of changing Bragg angle). The total counts along the entire length of the Bragg streak depends on its intersection with, and weighting by the pupil intensity function $|A(k_{\text{in}}){|}^{2}$, and for uniform illumination the counts can be simply normalized by the length of the streak. If the crystal is placed out of focus as depicted by Fig. 1(b), then the Bragg streak intensity is also modulated by the spatial profile of the diffraction efficiency of the crystal as discussed below in Sec. III B.

As mentioned above, Laue diffraction, which employs a collimated incident beam of broad bandwidth, also gives rise to many Bragg reflections. The pattern of these reflections is given in Fig. 2(c) for the same crystal and orientation as for the convergent-beam diffraction example in Fig. 2(a). In this case, the bandwidth was taken to be 25%, centered at a wavelength of 1 Å, for which there are 218 potential reflections to a resolution of 1.5 Å. As with convergent-beam diffraction, the reflections are fully integrated and the associated central wavelength for each reflection is only found after indexing. The intensities are modulated by the spectrum of the incident beam.

**FIG. 3.**
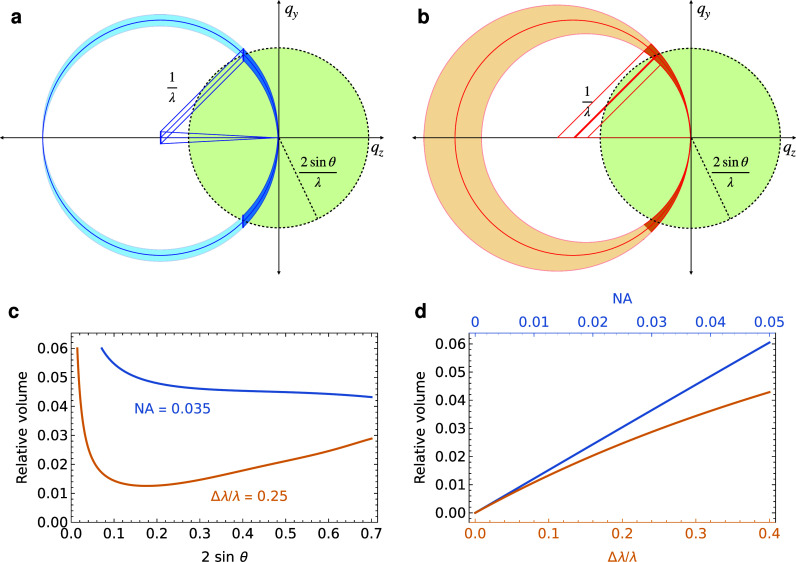
Convergent-beam diffraction patterns intersect large volumes of reciprocal space, comparable to high-bandwidth Laue diffraction. (a) The participating volume of reciprocal space in convergent-beam diffraction lies between Ewald spheres centered on the range of $-k_{\text{in}}$ vectors, shown shaded in blue. When considering diffraction extending to a maximum angle 2θ, only the region shaded in the dark blue participates. This volume is a fraction of the full volume of resolution-limited reciprocal space (indicated by the green shading), referred to as the relative volume. (b) Similarly, for Laue diffraction, the dark orange volume is the participating volume of reciprocal space for a given maximum scattering angle. (c) The relative volumes are plotted as a function of the resolution for NA=0.035 convergent-beam diffraction (blue) and for a relative bandwidth of 25% for Laue diffraction (orange). (d) The relative volumes for 2θ=45° ( 2 sin θ=0.7), plotted as a function of the lens NA or relative bandwidth.

It is perhaps surprising that the number of reflections in the convergent-beam diffraction pattern with NA=0.035 is comparable to the number of reflections in the Laue diffraction pattern with a bandwidth of 25%. Assuming that the density of reciprocal lattice vectors is relatively homogeneous throughout reciprocal space, the number of Bragg peaks or streaks in a pattern should be proportional to the reciprocal-space volume enclosed by the limiting Ewald spheres of incident beam angles or wavelengths. The volume covered by a 25% bandwidth is much greater than the volume addressed by a numerical aperture of 0.035, but this is only the case when the entire resolution limit is considered. The derivative of Bragg's equation shows that the equivalence of relative bandwidth and convergence angle depends on the Bragg angle according to

$$\frac{\Delta \lambda }{\lambda }=\frac{\Delta \theta }{\;\text{tan}\;\theta }$$ (3)

and so at low resolution, a small convergence angle is equivalent to a larger relative bandwidth than at a higher resolution. Figures 3(a) and 3(b) illustrate a cut through the *k_y_* - *k_z_* plane showing that when the comparison is limited to reflections at scattering angles 2θ<45°, or resolutions 1/d<0.77/λ, the volumes between the limiting spheres do appear comparable. Clearly, Laue diffraction provides a large participating Ewald volume at high resolutions in the back-scattering direction. The participating volumes for reflection are compared with the total reciprocal space volume for a given maximum scattering angle in Fig. 3(c) for convergent-beam diffraction and Laue diffraction. Here, the relative volume is defined to be equal to the ratio of the participating volume divided by the volume of a sphere of radius (2 sin θ)/λ. As such, for a given resolution, it is the fraction of reciprocal space that can be encountered in a single exposure. It is, thus, the reciprocal of the minimum possible number of exposures to measure a complete dataset at that resolution, assuming *P*1 crystal symmetry. Figure 3(d) plots the relative volume as a function of the numerical aperture or relative bandwidth for the two methods at a maximum scattering angle of 2θ=45°.

For completeness, a diffraction pattern recorded with a broad-bandwidth convergent beam will consist of Bragg patches rather than streaks. If the wavelength is varied, the Bragg angle of each Bragg streak in a monochromatic convergent beam diffraction pattern changes according to Bragg's law, sweeping the Bragg streaks in the radial direction of the diffraction pattern and moving the $k_{\text{in}}(\chi )$ deficit line across the pupil in a direction perpendicular to the line. Diffraction will occur as long as the deficit line falls within the lens pupil, so for a broad enough bandwidth each Bragg patch will be a mapping of that pupil and could provide a full topogram of a crystal placed out of focus (see Sec. III B). These Bragg patches may overlap, in which case they will tend to add incoherently since a given detector pixel would receive wave-vectors of differing wavelength.

### A. In-focus diffraction

When the crystal is placed in focus then all reflections originate from the same volume of the crystal, given by the overlap of the three-dimensional focal distribution with the volume of the crystal. Even if the focal spot is smaller than a unit cell of the crystal, the diffraction pattern will be indicative of the periodicity since the transverse component of the focal distribution a(x) is not strictly truncated. The focal spot for a perfect lens with a square pupil, for example, is described by a sinc function, and each plane wave component of the beam extends beyond the width of the focus. If the crystal is sufficiently thick, then the pattern will consist of Bragg streaks as depicted in Fig. 2(a). This is due to the periodicity of the crystal structure along the depth of the focus, which is visible when viewed back from the direction of the outgoing Bragg reflection.^45^ As the analysis above shows, the width of the Bragg streak (in the direction of increasing scattering angle 2θ) is proportional to the width of the reciprocal lattice peak in the *k_z_* direction, which itself is inversely proportional to the thickness of the crystal as seen in the projected view of the reflected beam. The intensity profile in this direction is the rocking curve of the reflection and so can be integrated to give a direct estimate of the structure factor, after first normalizing by any variation in the pupil function, $|A(k_{\text{in}}){|}^{2}$. This first requires knowing the pupil coordinates of the $k_{\text{in}}(\chi )$ vectors that gave rise to each Bragg streak, which itself requires indexing the pattern to solve for the crystal orientation. An approach for indexing such patterns was addressed by Gevorkov.^35^ Since the strength of diffraction is given by the product of the beam intensity (photons per area) and crystal volume, illuminating such a small volume of the crystal (within the focus) would demand a dose that is far beyond damage limits of a radiation-sensitive sample such as a macromolecular crystal. In-focus measurements of such samples are only practical by out-running damage with an x-ray free-electron laser pulse.

### B. Out-of-focus diffraction

A larger volume of the crystal can be exposed at lower dose if instead it is placed out of the beam focus. In the transverse plane at a defocus distance of many Rayleigh lengths (in the far-field of the focus), the beam is predominantly a diverging (or converging) spherical wave for which the ray direction $k_{\text{in}}$ is directly correlated with its transverse position as

$$\begin{array}{c} (x,y)=\frac{(k_{x},k_{y})\;z}{\sqrt{1/\lambda^{2}-k_{x}^{2}-k_{y}^{2}}},\\ =(\text{tan}\;\phi_{x},\;\text{tan}\;\phi_{y})\;z\approx (\phi_{x},\phi_{y})\;z. \end{array}$$ (4)

A crystal placed in this plane produces Bragg streaks that can therefore be mapped back to illuminating $k_{\text{in}}(\chi )$ lines that cut across this plane and hence across the face of the crystal as shown in Fig. 1(b). The intensity along a Bragg streak will be modulated by the diffraction efficiency of the crystal where it is intersected by the corresponding $k_{\text{in}}$ rays. Obviously, diffraction will not occur beyond the extent of the crystal. In a nondestructive experiment, rocking or translating the crystal will cause the intersection lines to sweep across the face of the crystal. A magnified diffraction topogram of the crystal can therefore be constructed from each Bragg streak, giving spatial maps of the diffraction efficiency of the particular Bragg reflections (Chufeng Li, in preparation). The magnification of the topogram is given by the ratio of the distance of the detector to the focus, divided by the defocus distance of the crystal. For defocus distances of about 10 *μ*m and a detector distance of 10 cm, the magnification can exceed 10 000, so that a 50 *μ*m wide detector pixel maps to an effective pixel size at the crystal of only 5 nm. As with projection imaging of non-periodic objects with high-NA lenses,^46^ the magnified topogram is equivalent to the defocused image that would be obtained of the crystal using a lens that forms a (defocused) image directly on a detector. In this case, the equivalence is to a dark-field image.^47^ The defocus gives phase contrast that may reveal lattice defects and the image can be simulated by the Fresnel transform of the projected diffraction efficiency of the crystal (projected along the direction of the incident beam).

Examining Fig. 1(d) shows it is necessary to rotate the crystal by an angle of at least 2α to sweep $k_{\text{in}}(\chi )$ across the full pupil, for a rotation axis that is perpendicular both to $q_{\text{hkl}}$ and the beam direction [e.g., around the *k_x_* axis in Fig. 1(d)]. Bragg streaks that are oriented parallel to the *k_x_* axis [such as the horizontal streaks in Fig. 2(a)] would be swept in the direction of *k_y_*. Bragg streaks located away from the *k_y_* axis may require larger rotation ranges to effect a full sweep, and streaks on the *k_x_* axis will not change position with a rotation about that axis. However, it can be seen that full topograms from a large number of Bragg reflections could be obtained from only a small rotation about a single axis.

A magnified topogram of the diffraction efficiency can also be obtained from a single snapshot pattern, albeit constructed from all available Bragg streaks in the pattern that are mapped back to their $k_{\text{in}}(\chi )$ positions (solved by indexing), such as shown in Fig. 2(b). The intensity profile of each Bragg streak is given by the product of the pupil function $|A(k_{\text{in}}){|}^{2}$, the two-dimensional map *C*(*x*, *y*) of the projected diffraction efficiency of the crystal, and the square modulus $|F(q_{\text{hkl}}){|}^{2}$ of the structure factor of that reflection. Obtaining precise estimates of the structure factors from a single pattern, thus requires normalizing by the pupil function and by *C*(*x*, *y*). Since the pupil function is known (from the direct beam, measured without a beamstop), it is possible to solve for *C*(*x*, *y*) and the structure factors. This is because, to a good approximation, the crystal contribution is common for all reflections that intersect a particular (*x*, *y*) position and the structure factor contribution is common for the entire Bragg line. The effect of any variation of the crystal diffraction efficiency can be thought of as being similar to a partiality factor, in that each reflection is generated by a different partial volume of the crystal. Solving for this partiality is a matter of finding self-consistent set of scalings *C*(*x*, *y*) and no additional model of the crystal structure is necessarily required (at least, not for static structure factors). However, the assumption that the crystal has uniform thickness and diffraction efficiency would imply the counts in each Bragg streak can be simply normalized by its length.

## IV. TIME-RESOLVED CONVERGENT BEAM DIFFRACTION

As mentioned in Sec. II, time can be encoded in a Laue diffraction pattern of a crystal using a chirped pulse, in which the wavelength varies with time.^18^ Since each Bragg peak in a Laue diffraction pattern selects a particular wavelength of the spectrum, with a chirped pulse each peak will therefore correspond to a particular arrival time on the crystal of the particular spectral component. That time can be determined by indexing the pattern, thereby solving for the wavelength of each Bragg peak. A particular volume of the crystal is exposed throughout the course of the exposure, so measurements at later times are from a structure that has received the prior cumulative exposure. Keith Moffat also suggested the use of monochromatic angularly chirped pulses, in which now the arrival time varies with the angle of incidence.^18^ Given that the indexing of a convergent-beam diffraction pattern determines the range of incident wave-vectors for each Bragg streak, it should be possible to encode and decode time in this case as well. While creating wavelength-chirped pulses requires particular operation of the XFEL facility or dedicated instrumentation,^48^ an angularly chirped pulse is produced naturally from a monochromatic pulse by using dispersive focusing optics. In the x-ray regime, these are either refractive or diffractive lenses.

We usually think of focusing by a lens as being a consequence of Fermat's principle of least time: rays all arrive in the focus by traveling along their shortest optical path. This is the case when considering the phase of the wave. The phase forms a spherical wavefront that converges to the focus. This wavefront, however, only coincides with the front of the pulse (or any propagating signal) for a lens without dispersion, which is to say one whose focal length is independent of wavelength. In a refractive lens, for example, the refractive index of the material, n=1−δ, is slightly less than 1 for X-rays and so the phase velocity in the lens, c/(1−δ), exceeds the speed of light in vacuum, *c*. A short pulse propagates through this material at the group velocity, given by $v_{g}=\partial \omega /\partial k=c/(n-\lambda \partial n/\partial \lambda )=c/(1+\delta )$, where *ω* is the x-ray frequency and we have assumed that the wavelength is far from absorption edges so that the refractive index decrement *δ* is proportional to $\lambda^{2}$. Rays that are deflected by the lens at different heights from the optical axis pass through different thicknesses *l* of the lens material so that the pulse front lags behind the wavefront by approximately 2δl. Given that the thickness of a refractive lens varies quadratically with the transverse height *h* from the optical axis (and deflection angle ϕ=h/f for a focal length *f*), it is found that this path difference depends on the square of the deflection angle as $L=\phi^{2}f$.^49^ The separation of the pulse front from the wave front for a diffractive optic such as a multilayer Laue lens^39^ has a similar dependence. Starting at the optical axis, each subsequent period (or bilayer) in a diffractive lens adds one additional wavelength of path so that rays diffracting toward the focus add constructively. Since the zone number increases with the square of the ray height, this extra path also increases quadratically with the lens height. Generally, the pulse front of a beam focused by a dispersive lens lags behind the phase front by

$$T_{X}(\phi )=-\frac{\phi^{2}f}{2c}\;V,$$ (5)

where

$$V=\frac{\lambda }{f}\frac{\partial f}{\partial \lambda }$$ (6)

is the dispersive power of the lens,^50,51^ describing the relative change in focal length for a relative change in wavelength. For diffractive lenses, the focal length is inversely proportional to wavelength and so *V* = −1. The quadratic dependence of *δ* on wavelength gives *V* = −2 for refractive lenses, twice the dispersive power of diffractive optics. As examples, a compound refractive lens stack with a radius of 500 *μ*m and focal length of 10 cm gives a maximum propagation time difference of 8 fs for rays arriving in the focus. Similarly, a typical high-NA multilayer Laue lens may consist of more than 10 000 layers, and since two lenses are used together to achieve two-dimensional focusing, the optical paths of rays reaching the focus exceed 20 000 waves. At a wavelength of 1 Å (12 keV photon energy), this is 2 *μ*m of path difference, causing the marginal ray to arrive in the focus 6.7 fs after the axial ray.

**FIG. 4.**
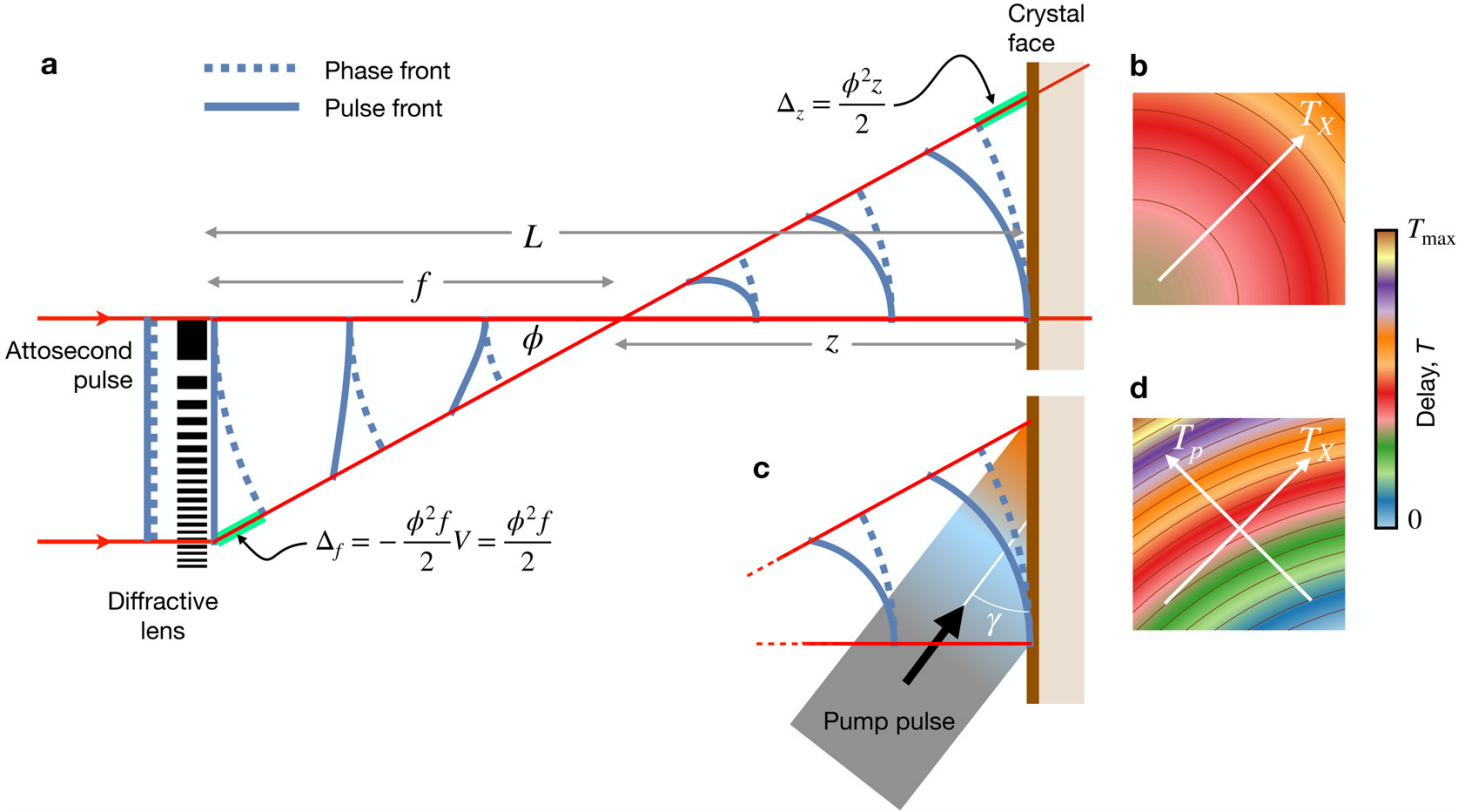
(a) A short collimated pulse focused by a dispersive lens lags behind the phase front by a time $\Delta_{f}/c$ that depends quadratically on the angle ϕ of the ray with the optical axis, shown here for a diffractive lens with a dispersive power of *V* = – 1. Rays, thus, arrive at the focus with this delay. For a flat crystal placed a distance *z* downstream of focus, the rays require an additional time of $\Delta_{z}/c$ to pass the face of the crystal. For a plane wave pump pulse that arrives simultaneously across the crystal face, the pump-probe delay *T* will vary with arrival time of the probe pulse on the crystal *T_X_*, given in (b). An inclined pump pulse (c), or one with a tilted pulse front, also maps arrival times *T_p_* to position, extending the range of delay times to $T=T_{X}-T_{p}$ (d).

When a crystal is placed at a distance *z* downstream of the focus, there is an additional path difference that contributes to the propagation time. As seen from Fig. 4(a), the path length of a ray propagating from the focus to the face of the crystal (a transverse plane), is z(1/ cos ϕ−1), so for small angles the angular chirp of the pulse arriving at the plane of the crystal becomes

$$T_{X}(\phi )=\frac{\phi^{2}}{2c}(z-V\;f).$$ (7)

Time ranges can therefore be increased by moving a crystal away from focus. Even achromatic focusing optics such as Kirkpatrick-Baez mirrors, for which *V* = 0, provide a temporal variation across the crystal when it is placed out of focus. The largest time range of the chirp that can be achieved will be limited by the NA of the lens (setting the maximum value of ϕ) and size of the crystal, since these will limit the range of angles of the diverging beam that intersects the crystal. An advantage of this scheme is that crystal volumes are not exposed by prior times of the chirp since rays of differing deflection angle (and hence different delay) intersect different volumes of the crystal.

A map of the arrival time of the $k_{\text{in}}$ wave-vectors at the crystal face is shown in Fig. 4(b) for an off-axis lens, with delay time shown as color. The convergent-beam diffraction pattern given in Fig. 2(a) shows how the incident $k_{\text{in}}$ rays of Fig. 2(b) map to the Bragg streaks of a crystal placed in focus (*z* = 0). A streak that is oriented with its long axis parallel to the direction of increasing time in the pupil map, thus, gives a sweep of time along the Bragg streak. The structure factor of that particular reflection can be extracted as a function of time by plotting the intensity along the streak. A streak oriented perpendicular to that will not have a significant variation in time, but will nevertheless correspond to particular arrival times. An on-axis lens, with its pupil centered on the optical axis (where the arrival time is the shortest), will give rise to Bragg streaks where time increases with distance in both directions from some particular point.

A dataset consisting of a full time sweep of the diffraction intensity at every reciprocal lattice point requires measuring enough patterns from crystals in different orientations. As compared with the number of crystals required to obtain a complete dataset irrespective of time, dependent on the participating volume per exposure plotted in Fig. 3(c), a greater number of exposures are required to obtain completeness at every time point. This can be crudely estimated by considering how many participating peaks are observed in a stopped-down pupil that covers a region of where the time is considered constant (i.e., within a time bin). Given the approximately linear dependence of volume on NA, as seen in Fig. 3(d), the number of required patterns, therefore, increases with the number of time-bins to which the data are apportioned.

## V. VELOCITY-MATCHING CONDITIONS

The changes in electron density to be measured may be initiated by a pump, such as a pulse of visible light, UV, or soft X-rays. Achieving a temporal resolution considerably below 1 fs requires that path lengths of both pump and probe beams are maintained to much less than 300 nm throughout the interaction volume of the two beams with the molecules in the crystal. This can easily be achieved when the crystal is thinner than about 300 nm or if the refractive indices of the crystal are approximately equal for the pump and probe pulses, such as with a soft x-ray pump. In that case, the pump and probe must propagate collinearly through the crystal volume.

The refractive index for a visible-light or UV pump will differ considerably from that for the x-ray probe, and consequently, these pulses will propagate through the crystal at different speeds. The refractive index for the x-ray probe, *n_X_*, will be approximately equal to unity, whereas the refractive index for the pump, *n_p_*, will likely be larger depending on the wavelength. For example, at visible wavelengths, the refractive index of a protein crystal may be larger than that of water, which is 1.33. In that case, the pump pulse propagates considerably slower than the probe and would lag the probe by 1 fs after propagating through a sample thickness of 1.2 *μ*m. Maintaining a fixed delay time along the path of the incident x-ray beam places constraints on the geometry of the crystal and on the arrangement of the pump and probe pulses.

**FIG. 5.**
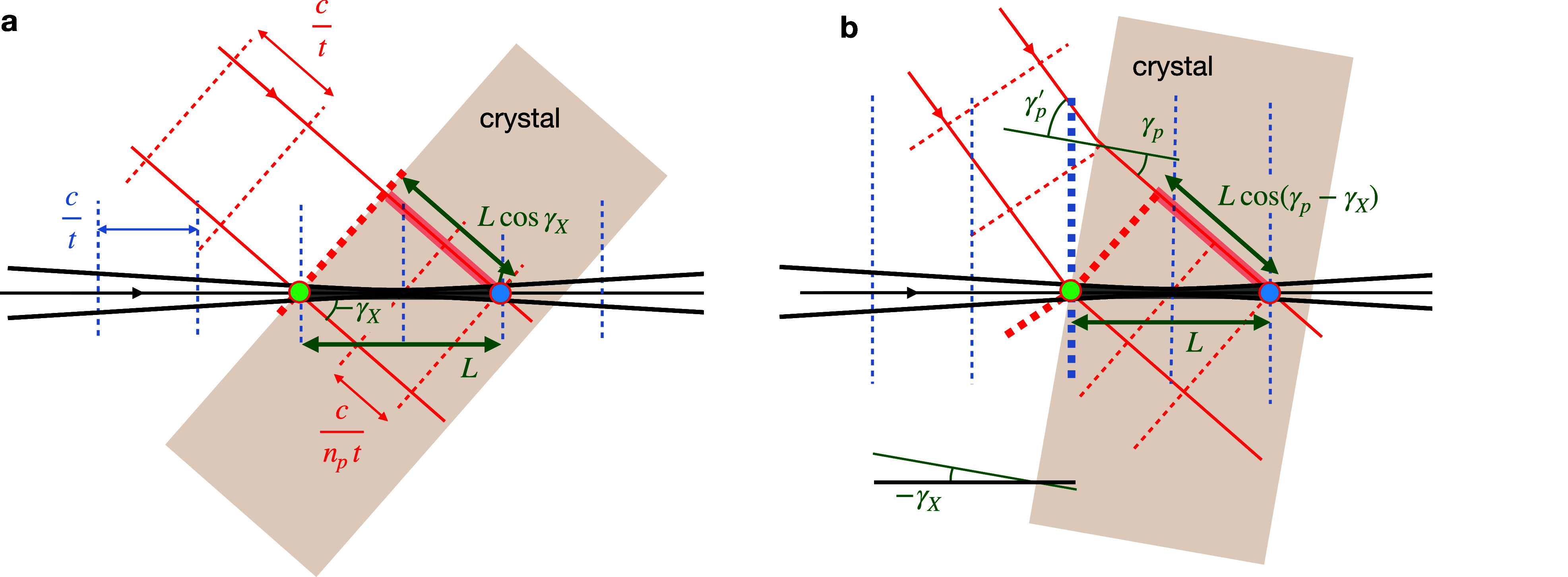
Orientations of the crystal, pump, and probe pulses required to maintain the delay between the pump pulse (red) and focused x-ray beam (black), along the path of the x-ray beam as it propagates through the crystal with a refractive index $n_{p}>1$ for the pump and *n_X_* = 1 for the probe. Phase fronts of the x-ray pulse and the pump pulse are indicated by blue and red dashed lines, respectively. No pulse tilt is assumed. (a) Pump pulse incident normal to the crystal face. (b) Pump pulse refracted by the crystal face.

With the crystal in the x-ray focus, this slippage in the case of *n_p_* > *n_X_* can be avoided by using a crystal with a flat face and tilted so the surface normal is inclined to the x-ray optical axis, as shown in Fig. 5(a) where the pump pulse impinges the crystal perpendicular to its face. The width of the pump pulse should be large enough to illuminate the full path of the focused x-ray beam in the crystal. Consider the case where the x-ray probe and pump pulses arrive simultaneously at the surface of the crystal, indicated by the green circle in the figure. Given that $n_{X}\approx 1$, the x-ray beam then propagates through the crystal medium at a speed *c* to arrive at a distance *L* after a time *L*/*c*, as indicated by the blue circle. This will intersect a different ray of the pump pulse, traveling with a speed $c/n_{p}$ but which only has to travel a distance $L\;\text{cos}\;\gamma_{X}$ in the same time. This is satisfied for all distances *L* when $\text{cos}\;\gamma_{X}=1/n_{p}$, which for $n_{p}=1.33$ (as an example) occurs at $-\gamma_{X}=41.2{}^{\circ}$. Note that when *n_p_* < *n_X_*, which may be the case for a soft x-ray pump, then the geometry must be reversed so that the crystal face is perpendicular to the x-ray beam and the pump tilted so that it travels a longer path in the crystal before intersecting the x-ray beam.

Figure 5(a) is not the only available geometry that can satisfy the velocity-matching condition, which can be achieved over a range of crystal tilts as illustrated in Fig. 5(b). In that figure, the pump pulse impinges on the face of the crystal at an angle and thus refracts, bending the light rays toward the surface normal. As before, the green circle indicates the point where the x-ray probe and pump pulses arrive simultaneously at the face of the crystal. At that point in time, the pulse front of the pump is distributed along the thick dashed line. The ray of the pump pulse that will ultimately meet up with the probe at the blue circle has at that time already propagated into the crystal and only need travel a distance $L\;\text{cos}(\gamma_{p}-\gamma_{X})$ in the time it takes the x-ray beam to travel a distance *L*. These two lengths are equal to the case in Fig. 5(a) (assuming the same refractive index, *n_p_*) and so the relative angle between the pump and the probe inside the crystal must be the same, requiring that the pump pulse is incident on the crystal at a steeper relative angle to the x-ray beam, assuming $n_{p}>1$. In this case, the velocity-matching condition is given by

$$\text{cos}(\gamma_{p}-\gamma_{X})=\frac{1}{n_{p}},$$ (8)

where *γ_p_* is the angle of the refracted pump beam relative to the surface normal. The incident angle, $\gamma_{p}^{\prime }$, is given by Snell's law, $\text{sin}\;\gamma_{p}^{\prime }=n_{p}\;\text{sin}\;\gamma_{p}$. As *γ_X_* is reduced to zero (with the crystal face perpendicular to the x-ray beam), the necessary incidence angle of the pump, $\gamma_{p}^{\prime }$, increases to a value given by $\text{sin}\;\gamma_{p}^{\prime }=\sqrt{n_{p}^{2}-1}$ with $\gamma_{p}^{\prime }=61.3{}^{\circ}$ at $n_{p}=1.33$. The crystal face can continue to be tilted beyond this angle in the positive sense so that both the pump and probe beams are incident on the same side of the surface normal until the limit is reached at $\gamma_{p}^{\prime }=90{}^{\circ}$, for which $\text{sin}\;\gamma_{p}=1/n_{p}$. For $n_{p}=1.33$, this is reached at $\gamma_{X}=7.5{}^{\circ}$.

Since the x-ray beam has some width, there will be a contribution to the error ΔT in the time delay *T* between the pump and probe pulses. For an x-ray beam width of *δ*, the difference in delay times between X-rays intersecting the same pump ray is given by

$$\begin{array}{c} \Delta T=\frac{\delta }{c}\left(\frac{n_{p}}{\;\text{sin}(\gamma_{p}-\gamma_{X})}-\frac{1}{\;\text{tan}(\gamma_{p}-\gamma_{X})}\right),\\ =\frac{\delta }{c}\;\sqrt{n_{p}^{2}-1} \end{array}$$ (9)

in the velocity-matching condition of Eq. (8). The beam intersecting the crystal reaches a maximum width of L NA for a crystal thickness *L*, assuming the beam is focused midway. Thus, for a crystal 3 *μ*m thick, this error reaches 0.3 fs for $n_{p}=1.33$ and NA=0.035, giving a mean error over the crystal thickness of 0.15 fs. The error decreases with decreasing *n_p_*, NA, or crystal thickness. Other examples are discussed below in Sec. VIII, following the analysis of the temporal resolution achievable when the crystal is placed out of focus to obtain magnified topograms.

The velocity-matching schemes using a crystal with a flat face are required when the refractive indices of the crystal medium differ significantly for the pump and probe pulses, which is the case for a visible or UV pump. In this case, all the crystals in a serial diffraction experiment must have faces parallel to each other, located on a larger flat surface that can be tilted to the x-ray beam and scanned in-plane to expose fresh crystals. Such samples could be obtained by various means, such as cutting slices of crystals, embedded in epoxy or a vitreous ice matrix, using a microtome or a focused ion beam, by exfoliation, or by molecular beam epitaxial growth on a substrate. One promising approach is to grow crystals in a volume confined by flat parallel membranes, as has been developed for electron diffraction.^52^

## VI. CROSSED-BEAM TOPOGRAPHY

While a time-resolved measurement can be made with the crystal placed in focus, using the angular streaking of $k_{\text{in}}$ wave-vectors mapped from the Bragg streaks to provide the temporal range and resolution, the diffraction entirely originates from the same volume of the crystal and so diffraction intensities corresponding to later times of the pulse may be perturbed by the earlier x-ray exposure. This is not the case when the crystal is out of focus, where the mapping of Bragg streaks to time is also a mapping to the position across the crystal face, and the probe pulse only exposes each diffracting volume for a time as short as the initial unfocused x-ray pulse. In this case, the velocity-matching conditions described in Sec. V still apply, and so if $n_{p}>1$, the pump pulse and the crystal must be tilted appropriately so that the delay is maintained as a particular x-ray propagates through the thickness of the crystal. This inclination then gives a variation of the time delay across the crystal face, which can be determined after mapping the Bragg streaks to the incident $k_{\text{in}}$ wave-vectors.

If *n_p_* = 1, then velocity matching demands that the pump and probe pulses propagate parallel to each other. In that situation, it is still possible to create a larger range of delays across the face of the crystal—by tilting the pulse front of the pump beam rather than tilting the direction of propagation. A linear pulse front tilt can be achieved by diffracting the pulse from a plane grating, for example.

A scheme of pump-probe topography using a visible pump and x-ray probe of different incident angles was first proposed for time-resolved diffraction measurements by Neutze and Hajdu,^20^ which they called crossed-beam topography. They proposed to use a very broad collimated x-ray beam and a large crystal, so that the spatial profile of the crystal is mapped directly onto the detector (without magnification) at each Bragg peak. For a typical detector pixel size of 75 *μ*m, millimeter-sized crystals are, therefore, required to achieve a reasonable number of time bins in a single measurement. A non-negligible bandwidth is required to ensure that the measured intensity is fully integrated and to avoid the high sensitivity to lattice distortions, which may cause significant intensity variations across the topogram. Our proposed use of a highly convergent beam, however, produces a greatly magnified topogram of the crystal that may be hundreds of pixels wide at the plane of the detector, even for micrometer-sized crystals, allowing hundreds of time bins in the dataset.

For a thin crystal, the topogram gives a map of the diffraction efficiency of the crystal projected along the incident x-ray beam direction.^53^ When the crystal face is tilted by *γ_X_* relative to the x-ray beam, Eq. (7) is modified to

$$\begin{array}{c} T_{X}(\phi_{x},\phi_{y})=\frac{z}{c}\{-\phi_{y}\;\text{tan}\;\gamma_{X}+\\ \phi_{y}^{2}\;{\text{tan}}^{2}\gamma_{X}+\frac{\phi^{2}}{2}\left(1-\frac{V\;f}{z}\right)\}, \end{array}$$ (10)

where $\phi^{2}=\phi_{x}^{2}+\phi_{y}^{2}$. In the general scheme of Fig. 5(b), the arrival time of the pump on the surface intersecting with the $k_{\text{in}}$ vector described by $\left(\phi_{x},\phi_{y}\right)$ is

$$T_{p}(\phi_{x},\phi_{y})=-\frac{z}{c}\frac{n_{p}\;\text{sin}\;\gamma_{p}}{\;\text{cos}\;\gamma_{X}}\left(\phi_{y}-\phi_{y}^{2}\;\text{tan}\;\gamma_{X}\right).$$ (11)

**FIG. 6.**
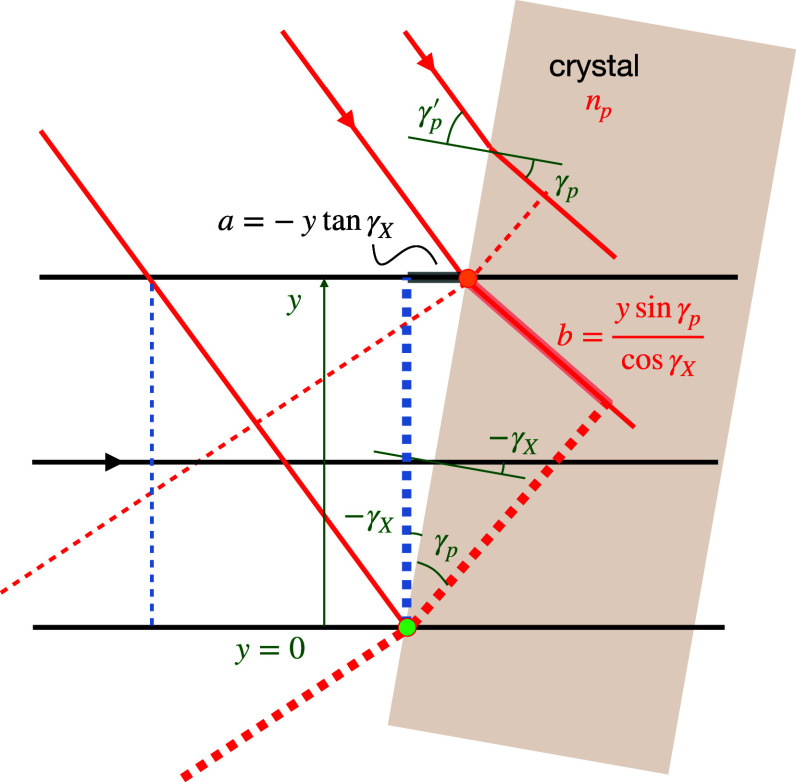
Geometry of crossed-beam topography for the same velocity matching conditions as Fig. 5(b), ignoring the convergence of the x-ray beam. The pump pulse (red rays) and x-ray pulse (black rays) coincide in time at the surface of the crystal at the green circle at a height *y* = 0. The x-ray pulse arrives later at the crystal at height *y* after a time $T_{X}(y)=a/c$, at which point the pump pulse has already traveled a distance *b* in the medium of refractive index *n_p_*, giving an arrival time of $T_{p}(y)=-bn_{p}/c$.

The geometry showing the linear sweeps of the two pulses across the face of crystal as a function of the transverse position $y=\phi_{y}\;z$ is given in Fig. 6. The pump-probe delay $T=T_{X}-T_{p}$ across the face of the crystal relative to the delay at the optical axis, without any additional pulse-front tilt, can then be written as

$$\begin{array}{c} T(\phi_{x},\phi_{y})=\frac{z}{c}\{-\phi_{y}\;G(\gamma_{X},\gamma_{p})(1-\phi_{y}\;\text{tan}\;\gamma_{X})+\\ \frac{\phi^{2}}{2}\left(1-\frac{Vf}{z}\right)\}, \end{array}$$ (12)

where

$$G(\gamma_{X},\gamma_{p})=\text{tan}\;\gamma_{X}-\frac{n_{p}\;\text{sin}\;\gamma_{p}}{\;\text{cos}\;\gamma_{X}}.$$ (13)

This term sets the magnitude of the linear component of the map of delays (here in the $\phi_{y}$ direction), proportional to the optical path differences highlighted in Fig. 6. It is seen that the linear component of *T* is equal to the time of flight across the transverse extent of the crystal ( $z\;\phi_{y}/c$) multiplied by the factor *G*, and as such may be the largest contributor to the range of time delays. It is found that for all incident pump angles $\gamma_{p}^{\prime }$, when the tilt *γ_X_* is set to obey the velocity-matching condition of Eq. (8), Eq. (13) reduces to a form similar to Eq. (9) so that

$$G(\gamma_{X})=\sqrt{n_{p}^{2}-1}.$$ (14)

Therefore, this linear component of *T* does not depend on the tilt of the crystal nor the corresponding inclination of the pump, as long as they satisfy the condition of Eq. (8). If an off-axis lens is used, such as depicted in Fig. 4, this linear term may add to or compensate the quadratic term caused by the pulse front curvature and defocus. Also, when *n_p_* = 1 then *G* = 0, showing that the tilt of the crystal does not affect the time delay in that case. Indeed, the shape of the crystal does not matter when the pump and probe both propagate through the crystal with the same speed, but the propagation direction of the pump must be set to be collinear with the x-ray probe. A linear variation in delay across the crystal face can instead be accomplished with a pulse front tilt of the pump or probe.

In the orthogonal transverse direction to the pump-pulse inclination, the variation in *T* is quadratic and spans a smaller range of times. Thus, the two-dimensional map allows both a large range in time delays as well as a dense sampling of these times. As an example, a map of delay times obtained with $\gamma_{X}=-41.2{}^{\circ}$ [as illustrated in Fig. 5(a) for $n_{p}=1.33$], f=1.25 mm, and z=0.3 mm is shown in Fig. 4(d). Here, the delay spans 50 fs.

As with the case of the crystal in focus, the range of tilts ϕ of the diverging x-ray beam will lead to errors in the velocity matching. A change in the angle ϕ of the ray relative to the optical axis can be thought of as a change of the inclination of the crystal *γ_X_*. It can therefore be compensated by adjusting the tilt of the pump pulse to maintain the velocity-matching condition of Eq. (8) by making the pump pulse slightly converging. This requires the defocus distance of the crystal to be much larger than the crystal thickness so that pump rays illuminating a particular x-ray path do not appreciably vary in angle.

Yet other pumping schemes can be made that use a linear pulse front tilt. For example, a constant delay across the entire overlap of the pump and probe beams throughout the crystal volume could be achieved when $n_{p}>1$ by adding a pulse front tilt to the x-ray beam to match the tilt angle of the crystal and illuminating it with a pump normal to the surface as in Fig. 5(a). This could be achieved with an asymmetric Bragg reflection from a flat crystal monochromator placed upstream of the lens. Both pulses would arrive simultaneously at the crystal face and then propagate with the same velocity component in the direction of the x-ray beam.

## VII. EXPERIMENTAL TEST—VITAMIN B_12_

**FIG. 7.**
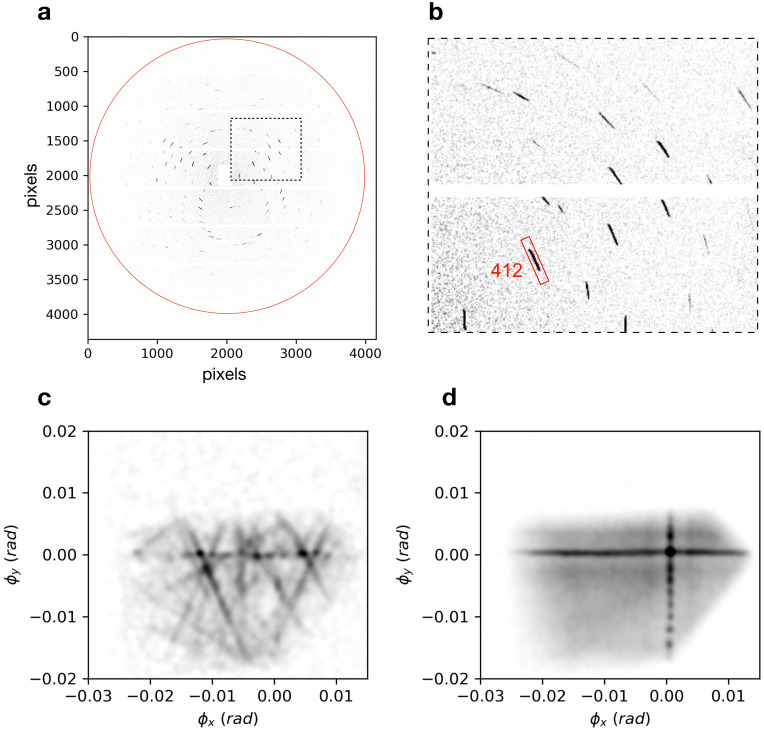
(a) Diffraction from a single vitamin B_12_ crystal placed 3.5 mm downstream of the focus of a 0.028 NA lens at a photon energy of 17.5 keV. The red circle indicates a resolution of 1 Å. The region outlined with dashed lines is shown enlarged in (b). (c) Map of the deficit lines obtained from the Bragg streaks of (a). (d) Map of the projected crystal diffraction efficiency obtained from integrating the 412 Bragg streak, highlighted in (b), over a 2.75° fine rotation scan of the crystal about the vertical axis.

A convergent-beam diffraction experiment was made using synchrotron radiation to demonstrate the mapping of Bragg streak intensities to a map of incident $k_{\text{in}}$ vectors. Figures 7(a) and 7(b) show a convergent-beam diffraction pattern measured from a vitamin B_12_ (cobalamin) crystal at a photon energy of 17.5 keV (wavelength of 0.7 Å). Measurements were conducted at the P11 beamline at the PETRA III synchrotron radiation facility using an x-ray microscope setup as previously described^46^ and an EIGER X 16M detector with a Si sensor (Dectris) consisting of 4150 × 4371 pixels. The detector was placed 18.3 cm from the sample, centered on the optical axis, to record a maximum scattering angle of 53° in the detector corner, corresponding to a maximum resolution of 1/(0.78 Å). A pair of on-axis MLLs of 0.028 NA were oriented orthogonal to each other to focus the beam in the horizontal and vertical directions with focal lengths of 1.25 and 1.26 mm, respectively. The lenses were prepared by masked deposition,^54^ and each consisted of 16 965 bi-layers with a minimum period of 2.06 nm (manuscript in preparation). As thick diffractive elements, each lens splits the beam into a focused order as well as an unfocused order.^39^ While a central obstruction of the lens paired with a pinhole just upstream of the focus can block all but the beam focused by both lenses, we instead used an unobstructed lens to ensure that full $k_{\text{in}}$ maps could be constructed from the Bragg streaks. A square pinhole of 10 *μ*m width was placed near the focus to block most of the flux in the zero order beam combinations, which at the focus form horizontal and vertical line foci and a collimated beam. The parts of these beams that passed through the pinhole illuminate the crystal placed downstream of focus and give rise to diffraction additional to the desired convergent-beam diffraction. This results in an increased diffraction intensity in the Bragg streak at the location (or locations) where the corresponding coordinate the of deficiency line cross the $\phi_{x}$ or $\phi_{y}$ axes. Far from this being a disadvantage, we found that the additional diffraction provides fiducials that assist in determining the coordinates of the incident deficit lines. The zero order component did require that a beamstop be placed on axis in front of the detector, however.

The vitamin B_12_ crystal was about 140 *μ*m in width and was mounted on a kapton mesh positioned at z=3.5 mm downstream of focus. The crystal form has orthorhombic *P* $2_{1}2_{1}2_{1}$ symmetry with unit cell parameters *a* = 15.70 Å, *b* = 22.15 Å, and *c* = 24.96 Å. The diffraction pattern in Fig. 7 was recorded for a single static orientation of the crystal with an exposure consisting of $2.5\times 10^{9}$ total incident photons. It is, hence, equivalent to one that may be obtained with an attenuated single pulse of an XFEL. This pattern was indexed using the known unit-cell parameters and a procedure related to that of the pinkIndexer^35^ as will be described elsewhere. The indexing solution provides the rotation matrix of the reciprocal lattice from which the reciprocal lattice vectors $q_{\text{hkl}}$ associated with each Bragg streak $k_{\text{out}}(\chi )$ can be determined. From each of these, the corresponding $k_{\text{in}}(\chi )$ deficit line can thus be obtained. The deficit lines cut across the lens pupil and hence also across the face of the crystal. A map of these deficit lines is given in Fig. 7(c), as a function of the angular components $\phi_{x}$ and $\phi_{y}$ of the $k_{\text{in}}$ vector [see Eq. (4)] and uncorrected for the lens pupil amplitude or structure factors of the reflections. It can be seen that this map is not complete—there are areas of the crystal that do not lead to strong diffraction—but it is clear that each point along every measured Bragg streak can be mapped to the particular $\left(\phi_{x},\phi_{y}\right)$ position in the pupil and hence to a particular x-ray arrival time *T_X_* (relative to the on-axis beam) as well as to the pump-probe delay time *T* if the pump pulse front is well characterized.

To evaluate the accuracy of the mapping of the Bragg streaks to their $k_{\text{in}}(\chi )$ deficit lines, we also collected a series of diffraction patterns where the crystal was rotated about the vertical axis over a range of angles comparable to the lens convergence 2α. In this case, most Bragg peaks sweep across the entire face of the crystal to form magnified topograms. We chose the intense 421 reflection, highlighted in the expanded pattern in Fig. 7(b), to generate such a topogram. The diffraction intensity of this streak, integrated over a fine rotation scan of 2.75° of the crystal is shown in Fig. 7(d), after isolating the streak from others in each frame of the rotation scan. (Accumulating directly on the detector leads to overlapping topograms from neighboring reflections.) In the figure, the crystal shape is more apparent than for that obtained from the still diffraction pattern, with a variation in diffraction efficiency corresponding predominantly to the projected thickness of the crystal.

A particularly striking feature of the intensity map of Fig. 7(d) is the cross pattern, formed from diffraction of the horizontal and vertical line foci and the collimated beam transmitted by the pinhole. These beams map to $\phi_{x}=0$ or $\phi_{y}=0$ and hence provide the absolute coordinate of the $k_{\text{in}}$ map from which the times *T_X_* can be determined to high accuracy. They provide fiducials that can be observed in single Bragg streaks and help to confirm the precision of the $k_{\text{in}}$ map of Fig. 7(c), where the horizontal line is particularly apparent. When two strong peaks of diffraction intensity are observed along a single Bragg streak, both $\phi_{x}$ and $\phi_{y}$ can be directly obtained. We find the accuracy of the mapping of the deficit lines in Fig. 7(c) to be better than a single pixel (which has an angular width of 0.4 mrad in this case). The crystal was well over-filled by the diverging beam which spans an angular range in both directions of 2α from −0.028 to 0.028 rad. As such, even longer Bragg streaks could have been measured with the crystal placed closer to focus.

We note that the diffraction intensity originating from $\phi_{x}=0$ or $\phi_{y}=0$ does not necessarily map back to the corresponding coordinate on the face of the crystal. For example, the collimated beam, which diffracts to the center of the cross, originates from the entire area of the crystal it illuminates (dependent on the size of the pinhole). Therefore, in a short-pulse measurement, the intensities in the cross cannot be used to obtain precise time-resolved data when used with a tilted pump pulse, but they do act as an in-built fiducial to define the $\phi_{x}$ and $\phi_{y}$ coordinates for each streak.

## VIII. TEMPORAL RANGE AND RESOLUTION

As mentioned in Sec. II, the temporal resolution achieved in an experiment depends on the durations of the pump and probe pulses as well as the timing uncertainties (jitter) between them. The pump pulse must necessarily be of short wavelength to reach sub-femtosecond duration. With current technologies, shorter wavelength pulses also give the best synchronization, achieved when pump and probe pulses of different wavelengths are generated from the same electron bunch in the XFEL accelerator.^19^ An EUV or soft x-ray pump, for example, can initiate electron dynamics by direct photoionization, providing opportunities to target specific atomic species as well as to directly study such processes as inter-atomic Coulombic decay^55^ and ultrafast charge transfer,^56^ providing complementary information to the dynamics of the ionization process itself.^12^ UV pulses have recently been generated with pulse durations less than 1 fs (Ref. 57). Nevertheless, these would be generated independently from the X-rays, resulting in a jitter that is usually not shorter than about 100 fs (Ref. 58). The temporal resolution can be improved by determining the relative delay of the two pulses on a shot by shot basis. This could be measured directly using a timing tool^59^ located downstream of the diffraction detector or upstream of the MLLs, requiring a calibration to account for path differences of the two pulses between the two measurement volumes.

As discussed by Moffat,^18^ the ability to map short intervals of time in the diffraction pattern does offer a way to accommodate timing jitter. He suggested to time the pump pulse to fall within the measurement interval so that the “time zero” could be identified. This strategy of identifying individual events in one timed measurement interval was used to determine the delay of the emission of a low-energy photoelectron relative to that of a higher-energy reference photoelectron from the same molecule.^12^ In that case, the emission time was encoded in the direction of the photoelectron, which was streaked using a circularly polarized IR pulse whose wavelength gave a 7.7 fs streaking interval recorded at 21 as/deg with an uncertainty of about 50 as. As detailed below, a similar range *T_m_* and resolution could be extracted from individual convergent-beam diffraction patterns, but measurements from many shots are needed to obtain complete data in reciprocal space and in time. The time span of a single measurement will likely be smaller than that desired for the experiment, which can be extended by stepping the relative delay of the pump and probe. However, due to timing jitter, the relative delay between the pump and probe will vary and be unknown shot to shot. Enough steps and shots must be acquired to cover the entire desired temporal range, with significant overlaps in times, so that the intensity of any given reflection at any given time point is sampled multiple times. Even if the timing jitter is larger than *T_m_*, we rely on enough diffraction intensities to be recorded to fill in all time points with a large number of overlaps. These overlaps provide the information to align the patterns onto a common time axis. We propose to carry this out using a correlation method, similar to the Expectation Maximization and Compression (EMC) algorithm^60^ that was created to determine particle orientations associated with single-particle diffraction patterns, but applied here to extract the underlying latent parameter of time (manuscript in preparation). Compared with the orientation problem (which relies on common lines of intersection in 3D reciprocal space), or the approach of nonlinear Laplacian spectral analysis to order Coulomb-explosion spectra in time,^61^ convergent-beam diffraction data from crystals provides a far greater degree of overlap between measurements.

For each snapshot diffraction pattern, the measured diffraction intensities of various reflections are mapped back to the corresponding incident $k_{\text{in}}$ wave-vectors in terms of the angular components $\left(\phi_{x},\phi_{y}\right)$ as illustrated in Fig. 7(c). Using the calculated mapping of $T(\phi_{x},\phi_{y})$ given in Eq. (12), intensities can be binned in time, with a sampling that depends on the range of delays spanned by $T(\phi_{x},\phi_{y})$ and the density of detector pixels.

When the crystal is placed in focus (*z* = 0), *T_m_* depends on the lens geometry as given by Eq. (5), proportional to ${\text{NA}}^{2}$ through the quadratic dependence on ϕ. As seen from Eq. (12), T increases linearly with defocus and consists of a linear term in ϕ due to the pump pulse and a quadratic term due to the divergent (or convergent) x-ray beam. The span *T_m_* is, thus, easily adjusted by changing the defocus, usually achieved in a serial crystallography experiment by moving the sample position in *z*. This range of times will only be achieved in a snapshot diffraction pattern if the exposed crystal is as large as the beam size.

Assuming velocity matching of the pump and probe pulses throughout the thickness of the crystal, the temporal resolution is dictated by how finely the map of *T* is sampled, which is given by the angular extent of the detector pixels. Since we aim for atomic resolution, the detector must be placed close enough to capture high scattering angles, which then places a limit on the smallest achievable pixel angular extent. Temporal resolution is, therefore, best maximized by using a detector with a large number of pixels and with the largest possible lens NA so that the Bragg streaks span as many pixel time bins as possible. In our vitamin B_12_ measurement of Sec. VII, the beam diverging from the focus, $A(k_{\text{in}})$, covered 140 × 140 pixels. If this experiment was implemented with XFEL pulses, the arrival times of rays in the plane of the crystal located 3.5 mm downstream of focus would span 12.4 fs. Due to the quadratic dependence of *T_X_* on ϕ, the temporal sampling is not uniform and is finer for earlier times. However, neighboring pixels may sample different times by less than 1 as the temporal resolution is determined by the range of arrival times of rays incident on the sensitive area of each pixel. These times vary most in the radial direction. In our example, neighboring pixels in the radial direction differ by 3 as at the early times for incident rays near to the optical axis [e.g., dark red portions of Bragg streaks of Fig. 2(a)] to 0.36 fs for the marginal incident rays [dark blue in Fig. 2(a)] and the mean resolution is 0.18 fs, as listed in column A of Table I. This corresponds to the time delay map for an on-axis collimated pump pulse with *n_p_* = 1 (e.g., a soft x-ray pulse). The table assumes the same detector size of 16 Mpixels as used in our measurement and which is available at the Bernina beamline of the SwissFEL.^62^

**TABLE I.** Temporal resolutions for different configurations, all with f=1.25 mm and NA=0.028, at a wavelength of 0.7 Å and with a centered 4000 × 4000 pixel detector positioned to record to a resolution of 0.78 Å at its corner and a pixel angular separation of 0.8 mrad. The time resolution ΔT is calculated as the variation in delays across the active area of a detector pixel. Column A: Experimental geometry used in Sec. VII; B: as A but with defocus; C: as B but with an off-axis lens; D: as B but with a crossed-beam UV pump.

	A	B	C	D
	Experiment	Near focus	Off-axis	Cross beam
*z* (mm)	3.5	0.18	0.18	0.18
Beam width (*μ*m)	200	10	10	10
Min. ϕ (rad)	0	0	0.014	0
Max. ϕ (rad)	0.04	0.04	0.091	0.04
*n_p_*	1.0	1.0	1.0	1.33
*T* (fs)	12.4	3.7	19.2	31.0
Min. ΔT (fs)	0.003	0.001	0.04	0.16
Max. ΔT (fs)	0.36	0.11	0.25	0.32
Mean ΔT (fs)	0.18	0.05	0.14	0.24

The 140 *μ*m vitamin B_12_ crystal used in Sec. VII is larger than required or is common for serial crystallography. Column B in Table I corresponds to a defocus of 180 *μ*m where the beam width is 10 *μ*m, suitable for micro-crystals and giving a 3.7 fs span of x-ray probe arrival times. For a given lens focal length, a larger range of x-ray arrival times can be achieved with an off-axis lens, as listed in the table as column C. Here, the bi-layer periods in the lenses were taken to decrease from 7.0 to 1.1 nm so that the deflection angles from each range from 0.010 to 0.066 rad. Along the diagonal of the pupil of the composite lens, the deflection angle ϕ ranges from 0.014 to 0.091 rad, giving a time span of 19.2 fs at the near-focus position.

Crossed-beam topography (with a UV pump pulse, for example) gives much greater control of both the range and resolution of times. In the velocity-matching condition, the linear contribution to *T* is proportional to the beam size at the crystal multiplied by the factor *G* given by Eq. (14), but this can be further adjusted by tilting the pulse front of this beam. Column D in Table I lists the timing characteristics for the on-axis lens configuration but with the pump pulse inclined to give a linear sweep of 30 fs. This produces a near-linear variation of the delay time across the $k_{\text{in}}$ map.

Other possibilities exist: for example, a quadratic dependence of arrival times of the pump beam could be achieved by focusing the pump beam with a dispersive lens, which could further reduce the range of delays across the beam and compensate for the variation of angles *γ_X_* due to the curvature of the diverging x-ray beam. In practice, the achievable resolution will be limited by how accurately Bragg streaks can be mapped back to the $k_{\text{in}}$ space of the pupil, which may depend on lens aberrations and changes in beam pointing, as well as the signal-to-noise ratio of the measurements. Our experimental tests indicate that the error is certainly less than 2 pixels, corresponding to the pixel sampling assumed in Table I.

## IX. CONCLUSIONS

Serial crystallography acquires many single-crystal snapshot diffraction patterns from randomly oriented and previously unexposed crystals as they are streamed or scanned across a pulsed x-ray beam. Here, we consider the potential for measurements in a pump-probe configuration to characterize transient valence electron densities at attosecond to femtosecond time scales and sub-ångström length scales for time-resolved quantum crystallography. Such measurements must necessarily be carried out with a high accuracy and performed on crystals that diffract to atomic resolution, which tend to have small unit cells that give rise to sparse snapshot diffraction patterns that have proved difficult to analyze. Dynamics must be initiated by a pulse of deep UV or shorter wavelength, with deep sub-femtosecond durations requiring soft x-ray pump pulses. The relative timing of the pump and probe pulses must be controlled or accounted for. As discussed in this paper, serial crystallography with a convergent beam, utilizing high-NA lenses such as MLLs, increases the control and precision for acquiring high-resolution diffraction data with sub-femtosecond temporal resolution. Convergent-beam diffraction patterns consist of Bragg streaks, rather than Bragg spots, which do not correspond to a single instant of time but map over a range of times that could span tens of femtoseconds, and which can be finely sampled with sub-femtosecond precision. A serial crystallography experiment could thereby be carried out by stepping the relative delay between the pump and the probe and collecting enough patterns to achieve a high completeness at any given time point (each structure factor measured several times, at least). We propose that timing jitter between the pump and probe pulses could be largely corrected by aligning time sequences of intensities obtained from single-shot diffraction patterns onto a common time line, by determining overlaps through correlations of intensity sequences.

In 0.1 fs, light only travels 30 nm. The wavefront control achieved by focusing a pulse to a nanometer spot, gained through the use of *in situ* wavefront sensing, is synonymous with path-length errors far below this length. Path-length errors could instead be introduced by the properties of the crystals. Any difference in the refractive index of the pump and probe will cause a walk off of the two beams as they propagate through a crystal, leading to an error of more than 1 fs in a crystal of thickness of 1 *μ*m and refractive index of 1.33 for a UV pulse. This can be avoided using thinner crystals or by inclining the pump and probe pulses to each other to ensure that the incident x-ray beam continues to intersect pump pulses with the same delay as they propagate through the crystal. This approach of crossed-beam topography demands crystals with a flat face and adds a linear component to the mapping of times to angular coordinates, encoded in each pattern. In contrast, the refractive index for a soft x-ray pump pulse will closely match that of the hard x-ray pump, requiring that the pump and probe propagate nearly collinearly through the crystal. In this case, if the pump and probe are generated from the same electron bunch, timing jitter will be considerably lower, appropriate for the shorter span of encoded times.

A potential drawback of convergent-beam diffraction, shared with Laue diffraction, is that the signal to background ratios of reflections are lower than for a collimated beam, since diffraction intensity is spread over many more pixels. This is a consequence of the much greater information content of the snapshot diffraction pattern. Nevertheless, as described in this paper, the use of high-NA x-ray lenses offers several compelling advantages for a broad range of serial crystallography experiments:

• The reciprocal-space volume that contributes to a snapshot diffraction pattern using high-NA X-ray lenses is much larger than attainable by Laue diffraction at XFEL sources, giving rise to a large number of reflections from small-unit-cell crystals that can be unambiguously indexed.
• Convergent-beam diffraction patterns consist of Bragg streaks that provide predominantly fully integrated intensities, further reducing the number of patterns required to obtain precise estimates of structure factors.
• By indexing the diffraction pattern, lines of incident wave-vectors $k_{\text{in}}(\chi )$ associated with each Bragg peak can be obtained. The entire set of these deficiency lines is bounded by the angular extent of the lens aperture.
• In a pump-probe experiment, the Bragg streak intensities encode time, which can be decoded using the coordinates of the corresponding deficiency lines. The span of times encoded in the Bragg streaks—and the corresponding temporal resolution—can be adjusted with defocus of the crystals.
• Bragg streaks are formed whether the crystal is placed in or out of focus. In focus, all diffraction originates from a common sub-volume of the crystal. Defocused, the deficiency lines cut across the face of the crystal. Any variations in crystal shape or efficiency can be accounted for in a single snapshot pattern by solving for the projection image of the crystal's diffraction efficiency.
• In addition to the two-dimensional focus, paired MLLs give rise to two orthogonal line foci and a collimated beam which can all illuminate the crystal to give in-built fiducials in the diffraction pattern that provide the absolute angular coordinates of the deficiency lines.

## Data Availability

Raw data were generated at the PETRA III synchrotron radiation facility. Derived data supporting the findings of this study are available from the corresponding author upon reasonable request.
